# Mechano-Enzymatic Deconstruction with a New Enzymatic Cocktail to Enhance Enzymatic Hydrolysis and Bioethanol Fermentation of Two Macroalgae Species

**DOI:** 10.3390/molecules23010174

**Published:** 2018-01-17

**Authors:** Sameh Amamou, Cecilia Sambusiti, Florian Monlau, Eric Dubreucq, Abdellatif Barakat

**Affiliations:** 1 UMR, Ingénierie des Agropolymères et des Technologies Emergentes (IATE), CIRAD, Montpellier SupAgro, INRA, Université de Montpellier, 34060 Montpellier, France; sameh.amamou@supagro.fr (S.A.); cecilia.sambusiti@gmail.com (C.S.); Florian.Monlau@Apesa.Fr (F.M.); Eric.Dubreucq@supagro.fr (E.D.); 2 APESA, Plateau Technique, Cap Ecologia, Avenue Fréderic Joliot Curie, 64230 Lescar, France; 3 AgroBioSciences, Mohammed VI Polytechnic University, Lot 660-Hay Moulay Rachid, Ben Guerir 43150, Morocco

**Keywords:** bioethanol, enzymatic hydrolysis, macroalgae, mechanical pretreatment

## Abstract

The aim of this study was to explore the efficiency of a mechano-enzymatic deconstruction of two macroalgae species for sugars and bioethanol production, by using a new enzymatic cocktail (Haliatase) and two types of milling modes (vibro-ball: VBM and centrifugal milling: CM). By increasing the enzymatic concentration from 3.4 to 30 g/L, the total sugars released after 72 h of hydrolysis increased (from 6.7 to 13.1 g/100 g TS and from 7.95 to 10.8 g/100 g TS for the green algae *U. lactuca* and the red algae *G. sesquipedale*, respectively). Conversely, total sugars released from *G. sesquipedale* increased (up to 126% and 129% after VBM and CM, respectively). The best bioethanol yield (6 g_eth_/100 g TS) was reached after 72 h of fermentation of *U. lactuca* and no increase was obtained after centrifugal milling. The latter led to an enhancement of the ethanol yield of *G. sesquipedale* (from 2 to 4 g/100 g TS).

## 1. Introduction 

Over the last decades, the world has been facing critical economic and environmental issues, such as the exhaustion of fuels, environmental pollution and climate change, combined with the increase of the world population. These issues led to the expansion of research and development on renewable and sustainable biofuels [1,2]. Both lignocellulosic biomasses and algae constitute sustainable sources of bioenergy and biomolecules (i.e., surfactant, bioethanol, biogas and biodiesel) and they represent promising alternative sources to petroleum-based fuels and chemicals.

In particular, macro- and/or micro-algae permit us to overcome the major limitations associated with lignocellulosic plants [3]. Macroalgae, also called seaweeds, represent renewable abundant biomasses, which could be easily cultivated in aquatic environment. Thus, they do not compete with land use, and water consumption necessary for terrestrial plants.

Furthermore, macroalgae are characterized by a higher growth rate than lignocellulosic biomasses and higher hydrolysable sugar contents than crops with almost no lignin [4,5,6]. Macroalgae are multicellular photosynthetic organisms divided into three major groups: green, red and brown algae, according to the thallus color derived from natural pigments and chlorophylls [4,7]. Generally, the amounts of carbohydrates vary between 25–60%, 30–60% and 30–50% dry wt. for green, red, and brown algae, respectively. In particular, green algae are mainly composed of mannan, ulvan, starch and cellulose, while red algae are mainly composed of carrageenan (up to 75% dry wt.) and agar (up to 52% dry wt.) as polysaccharides [6].

**Figure 1:**
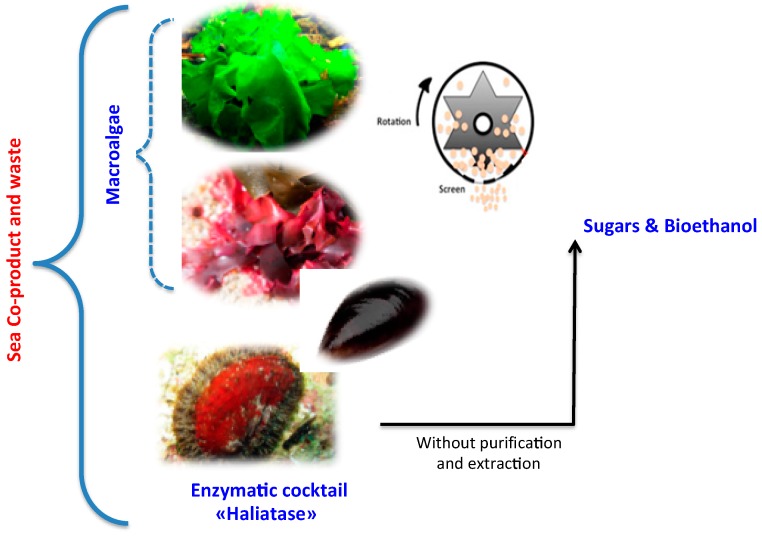
Mechano-enzymatic pretreatment and deconstruction of macroalgae developed in this study.

Several researches have reported the use of marine alga biomass as bioethanol feedstock. Different macroalgae groups such as *Gelidium amansii* [8], *Gracilaria salicornia* [9] and *Kappaphycus alvarezii* belonging to red seaweed and green algal species such as *Ulva* spp. have been considered as potential sources for conversion to bioethanol. As the interests in seaweeds were expanding, intense research was required for an efficient use of this biomass. However, it still faces technical and economic challenges and still depends on the development of eco-friendly pretreatment and conversion methods [10]. Since this step is often required to facilitate the enzymatic hydrolysis of macroalgae and their further sugars and bioethanol conversion. So far, the most common pretreatments used to enhance the hydrolysis and thus bioethanol production of macroalgae are physical (wet oxidation, thermal, milling and oven drying), chemical (acidic and alkaline), and thermo-chemical pretreatments [10]. However, one of the major drawbacks of using thermal and thermo-chemical pretreatments is the possible formation of organic acids and furan derivatives, which can inhibit bioethanol fermentation [11,12]. In addition, to find the most sustainable and cost-effective pretreatment, another challenge of producing bioethanol and interesting molecules from *G. sesquipedale* (red) and green *U. lactuca* (green) macroalgae is to find the specific enzymes able to efficiently hydrolyze their polysaccharides (Figure 1). Mechanical fractionation of biomass is one promising route that can contribute to a future sustainable dry biorefinery without water consumption and without waste production. Grinding or dry fractionation can be easily introduced in a biorefinery scheme improving the overall sustainability process [13]. Thus, coupling mechanical fractionation with enzymes is a promising biorefinery scheme of algae biomass valorization. In addition, the use of this natural enzymatic cocktail (i.e., Haliatase) coupling to mechanical fractionation has never yet been investigated in algae biomass biorefineries. Thus, the main objectives of this study were the following:

(i) Explore the efficiency of a new enzymatic cocktail to hydrolyze polysaccharides of two macroalgae species (red and green sp.)
(ii) Study the effect of two mechanical pretreatments, centrifugal milling (CM) and vibro ball milling (VBM) on enzymatic hydrolysis and bioethanol fermentation of the two-macroalgae species (red *G. sesquipedale* and green *U. lactuca*) (Figure 1).

## 2. Results and Discussion

### 2.1. Chemical Composition

The chemical compositions of both red *G. sesquipedale* and green *U. lactuca* macroalgae are shown in Table 1. *U. lactuca* had higher ash content (32 g/100 g TS) than *G. sesquipedale* (11 g/100 g TS). These values are in agreement with literature studies that reported ash values ranging from 11 to 34 g/TS and from 9 to 20 g/100 g TS, for green and red macroalgae, respectively [8,14,15]. Similar protein content (13 and 16 g/100 g TS) was observed for *U. lactuca* and *G.sesquipedale*, in accordance with literature data for red (10–16 g/100 g TS) and green (12–21 g/100 g TS) macroalgae [14,15,16].

Total sugar content of *U. lactuca* and *G. sesquipedale* were 25.8 and 30.9 g/100 g TS, respectively. Similar sugar content was reported by Jard et al. [16] for red and green macroalgae. As reported by Jung et al. [7], the red alga *G. sesquipedale* was mainly composed of glucose, galactose, and agar, while *U. lactuca* consisted of glucose, xylose, and rhamnose. Glucuronic and galacturonic acids were also detected in both algae with values of 5.01 and 3.32 g/100 g TS for the green and red algae, respectively. However, it is noteworthy that the chemical composition of macroalgae presents a great variability in the literature, which is related to several factors, such as species, geographical origin, season, environmental, and physiological variations, but also to the analytical method used for their characterization.

### 2.2. Particle Size of Macroalgae

**Figure 2:**
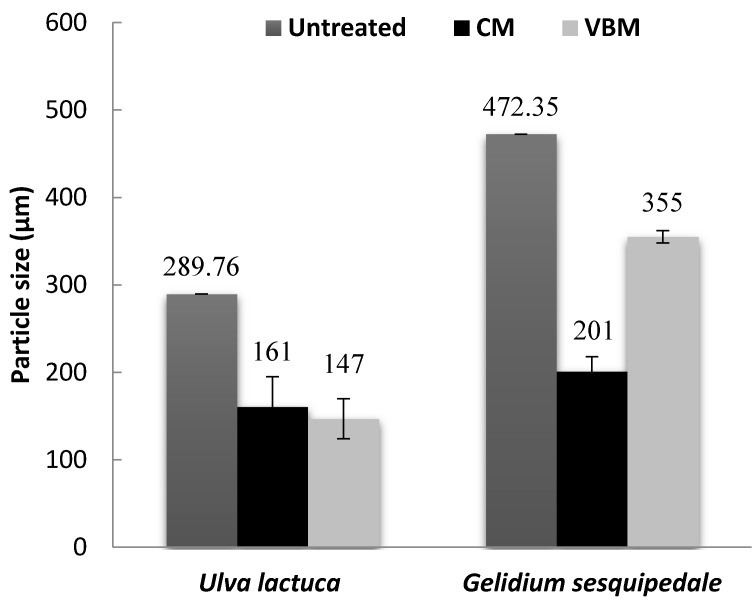
Mean particle sizes of untreated and milled algae biomass. Values correspond to mean ± SD (standard deviation) of measurement performed in duplicate.

Particle size of the untreated and milled macroalgae is reported in Figure 2. After milling, a lower mean particle size was obtained for the green alga *U. lactuca* (147–161 μm) than the red alga *G. sesquipedale* (201–355 μm), which can be explained by the lower particle size distribution of the untreated green algae biomass (289 µm) than the red one (472 µm). Furthermore, it was observed that for the red alga, CM was more effective than VBM in particle size reduction (Anova *p*-values < 0.05) (Figure 2); while for the green alga similar effect of VBM and CM was noticed with a slightly higher effect of VBM but not significant with an Anova *p*-values of 0.59. This could be explained by the high ash content (around 30%) and a possible synergistic impact between the mineral ash and the mechanical process (i.e., VBM) as previously mentioned by Motte et al. [13]. Indeed, they highlighted in their study that a mineral-vegetal co-milling in a VBM could significantly reduce the final particle size of the lignocellulosic biomass compared to a simple milling of lignocellulosic biomass.

### 2.3. Effect of Haliatase Cocktail Activity on Sugars Yield of Untreated Macroalgae

**Figure 3:**
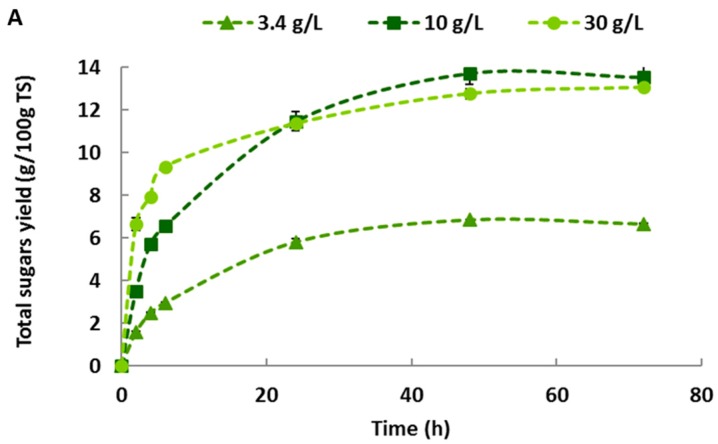

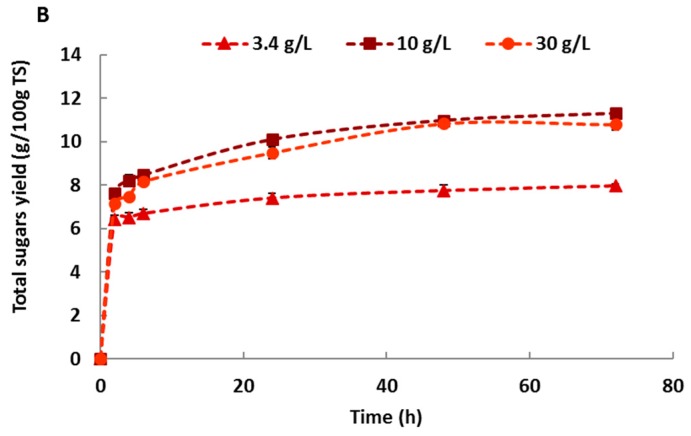
Total sugars released after 72 h of enzymatic hydrolysis for various enzyme dosages (3.4, 10 and 30 g/L) for both untreated (**A**) *Ulva lactuca* and (**B**) *Gelidium sesquipedale*.

The effect of Haliatase dosage on total sugars released during enzymatic hydrolysis of untreated *U. lactuca* and *G. sesquipedale* was investigated (Figure 3). For both macroalgae, the increase of the enzymatic concentration led to higher total sugars released. By increasing the enzymatic concentration from 3.4 to 30 g/L, the total sugars released after 72 h of hydrolysis varied from 6.7 to 13.1 g/100 g TS and from 7.9 to 10.8 g/100 g TS for *U. lactuca* and *G. sesquipedale*, respectively (significant difference with Anova *p*-values < 0.05) (Table 2).

However, the increase of enzyme concentration from 10 to 30 g/L did not lead to a further enhancement of total sugar yield of both red and green algae witch is assumed with Anova *p*-values of 0.13 and 1.55, respectively, for green and red algae. Finally, for both algae species, the major soluble sugar released during the enzymatic hydrolysis was glucose which is very pertinent in the case of ethanol production using the *Saccharomyces cerevisae* strain. Glucose released after 72 h of enzymatic hydrolysis varied from 5.8 to 10.5 g/100 g TS for *U. lactuca* and from 1.5 to 4.1 g/100 g TS for *G. sesquipedale* after increasing the enzymatic dosage from 3.4 to 30 g/L (significant difference with Anova *p*-values < 0.05) (Table 3). If the results of glucose yields were not significantly different from 10 g/L to 30 g/L for red algae (Anova *p*-values of 0.95), they were for green algae (Anova *p*-values < 0.05). Cho et al. [17] reported an enzymatic saccharification of *Gelidium amansii* using Celluclast 1.5 L (endoglucanase: (8.4 U/mL), Viscozymes L (β-glucanase: 1.2 U/mL) and a mixture of both enzymes. They obtained a glucose concentration of 5.5 g/L after an enzymatic hydrolysis using Celluclast 1.5 L. Interestingly, the glucose concentration released was improved to 7.6 g/L by using the mixture of “Celluclast + Viscozymes” enzymes after 48 h of hydrolysis. Nonetheless, in both assays, only the fibers rich in cellulose were hydrolyzed whereas agar was not solubilized [17].

### 2.4. Effect of Mechanical Pretreatments on Enzymatic Hydrolysis of Macroalgae

**Figure 4:**
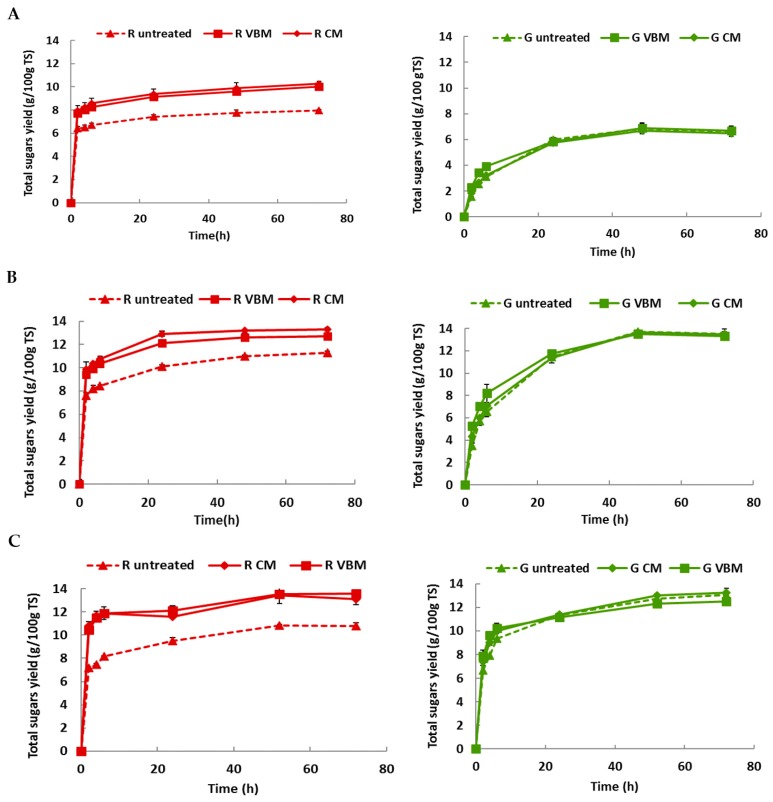
Total sugars released after 72 h of enzymatic hydrolysis for untreated and milled (G: Green, R: Red) macroalgae with an enzyme dosage of (**A**) 3.4; (**B**) 10 and (**C**) 30 g/L. Values correspond to mean ± SD of measurement performed in duplicate.

The effect of mechanical pretreatments (i.e., centrifugal milling, vibro-ball milling) on total sugars released during enzymatic hydrolysis of untreated *U. lactuca* and *G. sesquipedale* was investigated (Figure 4). The results revealed that, whatever the enzymatic dosage, mechanical pretreatments did not have any effect on the total sugars released from green alga *U. lactuca* (Anova *p*-values > 0.05) Conversely, total sugars released from *G. sesquipedale* increased after mechanical pretreatments (up to 126% and 129% after vibro-ball and centrifugal milling fractionation, respectively, Anova *p*-values < 0.05). Otherwise, fractionation was more effective in glucose releasing (up to 214% and 261% after vibro-ball and centrifugal milling, respectively). It is important to note also that mechanical fractionation seems to be less effective after increasing the enzymatic dosage from 3.4 g/L to 30 g/L. Thus, glucose released from *G sesquipedale* increased after CM by 161% and 62% for 3.4 and 30 g/L of enzymatic dosage, respectively. Furthermore, whatever the enzymatic dosage, CM was more effective than VBM in improving the total sugars and glucose released. Moreover, it is noteworthy that the high-energy requirement is one of the drawbacks of mechanical treatments. In a previous study, [18,19,20] reported that the energy requirement for CM (100 kWh t^−1^ TS) was lower than that of VBM (2000 kWh t^−1^ TS). Thus, CM was chosen for the performance of experimentation.

### 2.5. Bioethanol Fermentation of U. lactuca and G. sesquipedale

**Figure 5:**
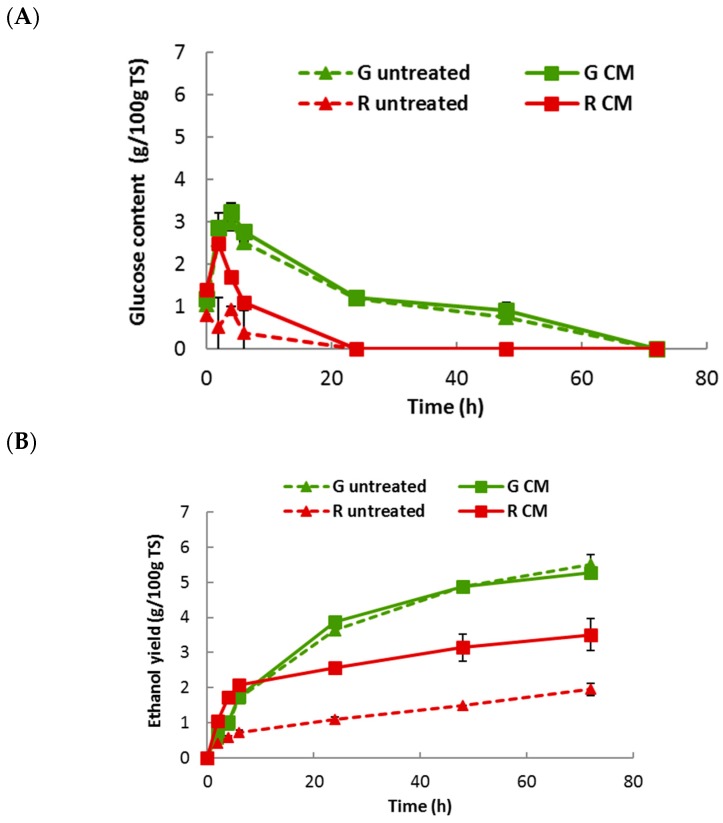
Simultaneous saccharification and fermentation of untreated and milled macroalgae (G: Green, R: Red) at an enzyme dosage of 10 g/L. (**A**) Glucose content (g/100 g TS); (**B**) Ethanol yield (g/100 g TS). Values correspond to mean ± SD of measurement performed in duplicate.

Taking into account the previous results, CM was chosen as mechanical treatment prior to a simultaneous saccharification and fermentation (SSF). Also, 10 g/L was selected as the optimal enzymatic dosage of Haliatase and, thus, used in saccharification process. Bioethanol yields of untreated and centrifugal milled macroalgae were evaluated and compared through SSF experiments (Figure 5). CM treatment did not affect the ethanol production of *U. lactuca* with values around 6 g_eth_/100 g TS (Anova *p*-values of 0.35), confirming the enzymatic hydrolysis data (Figure 4). Conversely, the CM led to an enhancement of the ethanol yield of the red algae (from 1.95 to 3.51 g/100 g TS, significantly different, with Anova *p*-values < 0.05). However, although bioethanol yield obtained with *U. lactuca* was higher than that of *G. sesquipedale*, the bioethanol conversion efficiency of the red one was higher, because the glucose content of red algae (9.62%) is lower than that (15.2%) of green algae (Table 1). Thus, after CM fractionation, 64% and 69% of bioethanol conversion efficiency (expressed in % of the theoretical yield) (Table 4) was obtained for green and red algae, respectively. Furthermore, for algae strains, the galactose and xylose were not consumed. Such observation could be attributed to a diauxic effect commonly observed but it is not totally satisfactory, as the galactose consumption of the red algae did not start even after a total depletion of the glucose, so probably 72 h would be too short to initiate diauxic effect since Berlowska et al. (2017) [21] Berlowska et al., 2017 have demonstrated that actually, *S. cerevisae* ethanol red was capable to metabolize galactose but in absence of glucose. Nevertheless, despite the fact that a large number of yeast species can metabolize xylose, only 1% of strains convert xylose to ethanol [22]. Thus, it is important to find the most active yeast species for bioethanol fermentation of hexoses (other than glucose) and pentose sugars, in order to achieve higher ethanol yield.

In this matter, Cho et al., 2014 [17], reported that glucose causes the repression of galactose uptake which decreased ethanol yield. The acclimation of galactose was then reported as the key of a fermentation process since it has allowed simultaneous utilization of glucose and galactose. In fact, ethanol yield doubled (from 0.21 to 0.44 g/100 g TS) after using *S. cerevisiae* acclimated to high concentration of galactose.

Regarding treatment effects on ethanol fermentation, Schultz-Jensen et al. [10] investigated the ethanol fermentation of the green macroalga *Chaetomorpha linum* after wet oxidation, hydrothermal treatment, plasma, and ball milling for 48 h at 40 °C. Interestingly, the best ethanol recovery was obtained after ball milling with an ethanol yield of 18 g/100 g TS, corresponding to 78% of the theoretical ethanol yield.

## 3. Materials and Methods

### 3.1. Macroalgae

Red alga *Gelidium sesquipedale* and green alga *Ulva lactuca* were obtained from the Morocco coast. Once collected, samples were washed with tap water and further air-dried (8% DM) and milled using a cutting mill to a particle size less than 2 mm (SM100 Retsch, Haan, Germany). Then, they were further milled using two equipments, characterized by different mechanical stresses, such as impact, compression, friction, and shear: (i) a centrifugal mill “CM” (Retsch ZM 200, Haan, Germany) with 0.25 mm screen size, operated at ambient temperature with a speed of 12000 rpm; (ii) a vibratory ball mill “VBM” (Retsch MM400, Haan, Germany) operated at ambient temperature, at a frequency of 15 s^−1^ for 5 min.

### 3.2. Enzymatic Cocktail

The enzymatic cocktail (i.e., Haliatase enzyme) has been obtained from KURA BIOTECH SPA, (Puerto Varas, Chile) and it is derived from the hepatopanchreas of cultured abalone (Haliotis rufescens). It is a multi-enzymatic cocktail capable of degrading the cell walls of macroalgae by hydrolyzing most of their polysaccharides components. It is composed of mainly β-glucanase (1875 U/g), carragenase (315 U/g) and agarase (440 U/g).

### 3.3. Enzymatic Hydrolysis

Enzymatic hydrolysis of untreated and milled samples was performed in 40 mL of closed flasks (working volume of 20 mL). An amount (1 g) of each sample (solid loading of 50 g/L), 2 mL of acetate buffer (500 mM) and 15.8 mL of ultra-pure water were added to each flask. The pH was then adjusted to 5.5 with NaOH (1 N) or HCl (2 N). Finally, 1.2 mL of sodium azide (final concentration 1 g/L) and 1 mL of concentrated Haliatase enzyme were added to have final enzymatic concentrations of 3.4, 10 and 30 g/L, respectively. Flasks were kept at 37 °C for 72 h with stirring 500 rpm. Samples were withdrawn at 0, 2, 4, 6, 24, 48 and 72 h and the corresponding supernatants were analyzed by HPLC (Waters corporation, Milford, CA, USA), equipped with a BioRad HPX-87H column (Biorad, Hercules, CA, USA) at 40 °C, a refractive index detector at 40 °C and a 0.005 M H_2_SO_4_ solvent at 0.3 mL/min. Tests were performed in duplicate, to evaluate the amount of C5-C6 sugars released during the enzymatic hydrolysis.

Sugar yields (g*_i_*/100 g TS) were calculated according to Equation (1):

(1) $${\mathrm{Sugar}\;“i”\;\mathrm{yield}}_{t}{\;=\;[C}_{t}\;\mathrm{sugar}\;“i”/C\;\mathrm{solid}]\;\times \;100$$

where: *C*_t_ sugar “*i*” (g_sugar “*i*”_/L) is the concentration of C5 and C6 sugars produced during hydrolysis, at time t; *C* solid (g TS/L) is the total solids concentration in the flask.

The analysis of variance (Anova) method was used to analyse the impact of the enzymatic dose and mechanical fractionation (CM and VBM) on both red and green algae, the confidence level considered was 95%.

### 3.4. Bioethanol Fermentation

Bioethanol yields of untreated and milled (CM) macroalgae were evaluated and compared through simultaneous saccharification and fermentation (SSF) experiments. Tests were performed by using unsterilized samples, into 40 mL flasks (working volume of 20 mL) closed with rubber septa and equipped with an air vent system, constituted of sterilized needle and filter, in order to evacuate the CO_2_ produced during the bioconversion. A lyophilized *S. cerevisiae* yeast strain (Ethanol Red^®^, FERMENTIS, a division of S. I. LESAFFRE, Lille, France) was used as inoculum. For this purpose, lyophilized cells were previously washed and then suspended in sterilized distilled water to a concentration of 30 g TS/L. Each flask contained: 1 g TS of sample (solid loading of 50 g/L), 1 mL of concentrated Haliatase enzyme, to have an enzymatic concentration of 10 g/L in each flask, 1 mL of yeast (30 g TS/L), 2 mL of nutrients, containing: 50 g TS/L yeast extract (Difco^®^), 4 g TS/L urea, 0.5 g TS/L chloramphenicol and 50 mM acetate buffer (pH = 5). Flasks were incubated at 37 °C for 72 h under stirring. Samples were withdrawn at 0, 2, 4, 6, 24, 48 and 72 h and the cell free supernatants were evaluated for ethanol and C6 sugars (i.e., glucose, galactose, fucose and rhamnose) concentrations by HPLC as previously mentioned.

Ethanol yields (g_ethanol_/100 g TS) were calculated according to Equation (2):

(2) $${\mathrm{Ethanol}\;\mathrm{yield}}_{t}{\;=\;[C}_{t}\;\mathrm{ethanol}/C\;\mathrm{solid}]\;\times \;100$$

where *C*_t_ ethanol (g_ethanol_/L) is the concentration of ethanol produced during SSF, at time t; *C* solid (g TS/L) is the total solids concentration in the flask.

The analysis of variance (Anova) method was used to analyze the impact of mechanical fractionation (CM and VBM) on both red and green algae bioethanol fermentation, the confidence level considered was 95%.

### 3.5. Analytical Determinations

Particle size distribution of untreated macroalgae was determined by a vibratory sieving apparatus (Analytical Sieve Shaker AS 200, Retsch^®^, Haan, Germany) equipped with six sieves of different sizes (1, 0.8, 0.71, 0.56, 0.32 and 0.2 mm). Particle size distribution of milled macroalgae was analyzed by a laser granulometry (MASTERSIZER 2000, Malvern Instrument, Orsay, France). Total Solids (TS), Volatile Solids (VS) and ash contents were determined according to APHA methods [22]. Ultimate analysis (C, N, H and S) was accomplished with an elemental analyzer (Elementar “VarioMacroCube”, Elementar group, Langenselbold, Germany). Proteins content was estimated by multiplying N by 6.25. Carbohydrates and uronic acids were determined according to a reduced scale hydrolysis procedure, based on the NREL Laboratory Analytical Procedure [23]. Briefly, 80 ± 1 mg of milled algae biomass was subjected to a two-stage sulfuric acid hydrolysis (1 h at 30 °C in 72 wt % H_2_SO_4_, followed by 1 h at 121 °C in 4 wt % H_2_SO_4_ for red algae and 3 h at 120 °C in 6 wt % H_2_SO_4_ for the green algae). Samples were withdrawn at 1 h, 2 h and 3 h and the cell free supernatants were evaluated for sugars (i.e., glucose, xylose, arabinose, galactose, fucose, rhamnose) and uronic acids (galacturonic acid and glucuronic acid) concentrations by high-performance liquid chromatography by HPLC system (Waters corporation), equipped with a BioRad HPX-87H column at 40 °C, a refractive index detector at 40 °C and a 0.005 M H_2_SO_4_ solvent at 0.3 mL/min. It is noteworthy that analytical determinations were performed in duplicate.

## 4. Conclusions

A comprehensive study was performed on the efficiency of a new natural enzymatic cocktail to hydrolyze polysaccharides of two types of seaweeds and produce bioethanol. The effects of two mechanical pretreatments were also tested for increasing bioethanol fermentation. The most effective enzymatic dosage for the saccharification process of green *U. lactuca* and red *G. sesquipedale* was 10 g/L and the highest values of glucose released were obtained with green algae after 72 h of enzymatic hydrolysis. Centrifugal milling was more effective in hydrolyzing red *G. sesquipedale* compared to vibro-ball milling, while the mechanical pretreatments applied did not show any effect on green *U. lactuca*. However, green *U. lactuca* showed the highest bioethanol yield compared to red *G. sesquipedale.*

## Figures and Tables

**Table 1:** Chemical composition of red *G. sesquipedale* and green *U. lactuca* Values correspond to mean ± SD (standard deviation) of measurement performed in duplicate.

Composition	*G. sesquipedale*	*U. lactuca*
TS (g/100 g FM)	91 ± 0	90 ± 0
VS (g/100 g TS)	78 ± 0.1	62 ± 0.7
Ash (g/100 g TS)	11 ± 0.3	32 ± 0.7
C (g/100 g TS)	34.7 ± 0.5	26.8 ± 1.5
N (g/100 g TS)	2.5 ± 0.1	2.3 ± 0.4
H (g/100 g TS)	5.7 ± 0.05	4.5 ± 0.6
S (g/100 g TS)	2.1 ± 0.02	3.7 ± 0.12
Proteins (g/100 g TS)	19.9 ± 0.82	15.9 ± 1.91
Total Sugars (g/100 g TS)	30.9	25.8
*Monomeric sugars* *		
Glucose (g/100 g TS)	9.6 ± 0.06	15.2 ± 1.01
Galactose (g/100 g TS)	20.3 ± 0.78	n.d.
Arabinose (g/100 g TS)	0.9 ± 0.06	n.d.
Xylose (g/100 g TS)	n.d.	3.1 ± 0.18
Rhamnose (g/100 g TS)	n.d.	7.5 ± 0.13
Fucose (g/100 g TS)	n.d.	0.5 ± 0.04
Glucuronic acid (g/100 g TS)	0.3 ± 0.03	3.86 ± 0.01
Galacturonic acid (g/100 g TS)	3.0 ± 0.06	1.15 ± 0.00

n.d.: Not detected; * Monosaccharide profile of polymeric carbohydrates determined after acid hydrolysis and HPLC quantification; SD: standard deviation.

**Table 2:** Total sugar yields (g/100 g TS) obtained after 72 h of enzymatic hydrolysis of untreated and milled algae with an enzyme dosage of 3.4, 10 and 30 g/L.

Samples	Enzyme Loading	3.4 g/L	10 g/L	30 g/L
Green alga: *Ulva lactuca*	Untreated	6.66 ± 0.04	13.52 ± 0.51	13.05 ± 0.16
	Centrifugal milling	6.48 ± 0.40	13.46 ± 0.61	13.24 ± 0.37
	Vibro ball milling	6.70 ± 0.35	13.33 ± 0.37	12.49 ± 0.20
Red alga: *Gelidium sesquipedale*	Untreated	7.96 ± 0.09	11.30 ± 0.13	10.79 ± 0.26
	Centrifugal milling	10.28 ± 0.06	13.28 ± 0.19	13.09 ± 0.48
	Vibro ball milling	10.03 ± 0.20	12.70 ± 0.11	13.59 ± 0.34

**Table 3:** Glucose yield (g/100 g TS) obtained after 72 h of enzymatic hydrolysis of untreated and milled algae with an enzyme dosage of 3.4, 10 and 30 g/L.

Samples	Enzyme Loading	3.4 g/L	10 g/L	30 g/L
Green alga: *Ulva lactuca*	Untreated	5.78 ± 0.00	12.45 ± 0.47	10.49 ± 0.10
	Centrifugal milling	5.79 ± 0.52	12.59 ± 0.50	10.71 ± 0.13
	Vibro ball milling	6.01 ± 0.60	12.63 ± 0.46	10.21 ± 0.08
Red alga: *Gelidium sesquipedale*	Untreated	1.48 ± 0.04	4.07 ± 0.22	4.12 ± 0.13
	Centrifugal milling	3.87 ± 0.17	7.09 ± 0.22	6.68 ± 0.70
	Vibro ball milling	3.17 ± 0.07	5.45 ± 0.10	6.35 ± 0.58

**Table 4:** Ethanol yield (g/100 g TS) obtained after 72 h of SSF of untreated and milled algae with an enzyme dosage of 10 g/L.

	Ethanol Yield (g/100 g TS)		Ethanol Efficiency (% Theoretical Yield *)	
Samples	Untreated	Centrifugal Milling	Untreated	Centrifugal Milling
*Ulva lactuca*	5.51 ± 0.29	5.27 ± 0.02	67.2%	64.3%
*Gelidium sesquipedale*	1.95 ± 0.17	3.51 ± 0.46	38.2%	68.7%

* Theoretical ethanol yield: 8 g/100 g TS and 5 g/100 g TS for *U. lactuca* and *G. sesquipedale* respectively.

## Abbreviations

VBM	Vibro-Ball Milling
CM	Centrifugal Milling
TS	Total Solids
VS	Volatile Solids
SSF	Simultaneous Saccharification Fermentation
